# The Pathology of Sleep
*Sleeplessness. Article Sleep. Dict. Prac. Med. By Dr. Copland. 1851.


**Published:** 1852-01-01

**Authors:** 


### Art. IV.—THE PATHOLOGY OF SLEEP.*

Every abnormal function or condition, whether it be of quality or quantity, is, of course, a pathological condition, as it is not health. This applies equally to psychical and physical states.

If we cannot logically term a total absence of a function a *disease* of that function, but rather of the organ which fulfils it, we may yet be justified in applying the term "Pathology of Sleep" not only to those prototypes of the somnolent state which indicate a deranged or morbid condition of the mental organ, but also to that negative state which we term insomnium or sleeplessness, a derangement or disorder of quantity, as the other states are of quality; so far the definition of the learned author of the "Medical Dictionary" is correct—"a non-recurrence or interruption of sleep."

Slumber, or healthy sleep, is a state of perfect intellectual abeyance, a fallow of the mind: in the words of Dugald Stewart, "A total suspension of volition as regards its influence over mental and corporeal faculties." It is thus a blessing with which the Creator has endowed the brain, which, during this unconscious repose, regains the power or energy of which the exertion of its natural faculty had deprived it: a *recollecting*, as Liebig would imply, of that living tissue which had been wasted or exhausted by exertion. Perfect, or sound sleep, then, is a healthy state. Hence we decidedly object to the assertion of Dr. Wilson Philip, that sleep indicates the imperfection of our nature.

The morbid state of weariness does in itself indicate imperfection; but sleep, its remedy, displays rather the perfection of our being, ministering so far to our happiness that we thus enhance our abstract fruition by the force of contrast.

The term sleep has been so often erroneously applied to its affinities or analogies, as to have imparted much discrepancy to the pages of psychology. Its physiology, also, has been often sadly perverted: M'Nish, for instance, terms it "an intermediate state between life and death."

Still there is no wonder that in contemplating this deep sleep, even "tired nature's sweet restorer" should, in darker ages, have been deemed the type of death—"*mortis imago et simulacrum;*" or that the Lacedemonians were wont to place the statues of Mors and Somnus together. No wonder that the poet should have made the first deep sleeper thus express himself—

---

* Sleeplessness. Article Sleep. Dict. Prac. Med. By Dr. Copland. 1851.

> "There gentle sleep
> First found me, and with soft oppression seized
> My drowned sense, untroubled, though I thought
> I then was passing to my former state,
> Insensible, and forthwith to dissolve."

The good Sir Thomas Brown was so deeply impressed with this likeness that "he did not dare to trust it without his prayers."

Another mind, however, equally pious, thus welcomes its visitation—

> "*Somne levris, quanquam certissima Mortis imago,*
> *Consortem cupio te tamen esse tori.*"

The more our mental repose, sleep, resembles the slumber of healthy children—we may indeed say the sleep of plants—an exhaustion of consciousness—the more will be its salutary refreshing of the brain, "a repair of waste, a due repose for past action."*

Now, whether the brain be exhausted by labour, excitement, or disease, sleep is the only mental prophylaxis of mental decadence, the only refresher of the spirit. It is, indeed, not only a potent remedy, but a faithful harbinger in disease. In fever, and other acute disorders characterized by insomnium, our first ray of hope is the coming on of a quiet sleep. In delirium tremens, to use the words of the late Dr. Mackintosh, of Edinburgh, it is "sleep or death."

In the physical indulgences of advanced life, sleep must be constantly ensured, or apoplexy will be a frequent consequence. Sleep is, therefore, a *remedy* of the deepest value, and although so much resembling death, is, indeed, its antithesis and prophylaxis. We may yet, thus far, admit something of an analogy between them. As we may say that we are always dying or approaching towards death, from the moment that we begin to live, so we are always *going* towards sleep directly after we wake and begin to exert our intellectual faculties.

The contrast of sleep is the state of waking; a condition not, of course, essentially morbid within certain limits, as it is the natural and active state of mind. Insomnium is only to be considered in a pathological sense, when, in consequence of wear of mind or body, there is a *necessity*, a *disposition*, to sleep, and yet it cannot be wooed to the pillow. A state of pervigilium, or watching, cannot with impunity be extended beyond eighteen or twenty hours, except in the mindless, thoughtless state of mania. If the mind, moreover, has been in energetic action, the necessity for repose becomes the greater; but if it be slothful, as that of the idiot, there is less need of sleep: the melancholic has lived forty days and nights without sleep. But when the power of sleeping is *often* suspended or impeded, insomnium becomes a very perilous disorder. It were not, indeed, too bold to affirm, that beyond this period, the state of waking or vigil is essentially that of derange-

* Copland.

ment. It may be at first so slight as to be scarcely if at all perceptible, as we may have so slight a bodily disorder that we are not aware of it: still the light disorder shall soon lead to organic disease, as protracted pervigilium may lead to confirmed insanity.

Now, if there be a sudden onset or stealthy approach of eccentricity (not that original or habitual eccentricity which constitutes character), combined with protracted pervigilium, our suspicion as to the health of the mind should instantly be aroused. These are most important moments; active and watchful management is imperative. For, as about ninety per cent. of those treated within the first three or four months recover, and as the prospect of cure decreases in a direct proportion as the months or years increase, so insomnium, as a primal symptom of insanity, is one of deep interest, and we must think the learned author of the "Dictionary" remiss in not having made a special allusion to the point. It is to this end, chiefly, that we have made the subject of sleep prominent in some of our later pages. This protracted insomnium is the result of an immediate physical cause which thus becomes of moment, because acting on important tissues, even as an ulcer on the palpebræ would, on healing, leave the eyelid useful as before, but if occurring on the cornea would impair or destroy the sense of vision. When, therefore, erethysm affects a brain, a thought, born of this unhealthy organ, constantly, incessantly, acting on its tissue like a rolling snow-ball, increases the degree of oppression, and thus resists remedy. This is the secret which explains the apparent paradox that sleeplessness is both a cause and effect of insanity, a sort of self-multiplication from one poisoned germ of thought.

Undue sleeplessness is at first a negative disorder, as it is the privative of sleep, whether we consider it a passive state, or, with Blumenbach and Cabanis, a real *function*. But fresh and unhealthy actions are directly set up, and it soon becomes a positive disorder.

Now, when we have defined true sleep, and reflect on its psychical essence of manifestations, we must, of course, include in the term all its prototypes, inasmuch as each, being abnormal, or in excess, is an indication of the disturbance of the healthy condition of the mind. Hippocrates thus refers to this excess both of sleep and its privative—

*Υπνος αγρυπνιη αμφοτερα του μετριου*
*Μαλλον γινομενα κακον.*

*Reverie* is the lightest form, an abstraction or intellectual concentration, and is often the half-sleep, or dozing time, following perfect insomnium. It is, indeed, far more waking than sleeping, and yet the wonderous stories are common as household words, how Mackenzie parodied, and Voltaire and La Fontaine versified, and Condorcet solved abstruse problems, and Haycock preached eloquent sermons, and above all, Tartini composed his exquisite "Sonata di Diavolo," and all this in sleep. But sleep it is not, any more than is that state which is darkened or brightened by a dream, or in which somnambulism plays its pranks, and in which we do not stop our ideas to reflect on them, as in waking reverie. Incubus is next, as it is marked by a temporary loss of power, yet volition (or will) is present. Somnambulism is the complete converse of this, as there is an obedience of action to volition, and yet the senses are passive. In the dream, while the senses are uninfluenced, volition is not obeyed. If we are tickled, we draw up the foot, but do not often wake; so, indeed, we endeavour, unconsciously, to relieve *malaise* of congestion by altering our position, yet then we may not wake. We say unconsciously, for sensation does not cease; if it did, we should cease to breathe, for the sensitive principle affects the lungs in sleep. The deep sleep of carus, catalepsy, trance, coma, apoplexy, stupor, syncope, asphyxia, are in themselves real disorder, the senselessness being but one symptom of the malady.

Allied to these is the *apparently* deep sleep of a condemned creature before execution—the effect of a *stunned* or paralysed brain. At these varied states we merely glance *en passant*, our chief subject being that form of stubborn wakefulness resulting from morbid activity or continued exertion of brain, which does not yield to the desire for sleep.

Now were it not for the working of mind in the brain, sleep would inevitably ensue, as a physical law, whenever the systemic power was exhausted or reduced to a certain point, even as the plant will sleep on the withdrawal of its stimulus. But if a thought on an interesting subject arises in the mind, the power of multiplying its kind, which is the characteristic of thinking, will not only keep the mind wide awake, but, from sympathy and the force of volition, will exert the same influence over the body, inducing restlessness or a frequent change of position.

If any long-continued strain on the imagination, or intense thought has been indulged in, then this insomnium is thereby increased, for the time of instinctive repose has passed, and disorder has set in. The associations of ideas do not cease with the voluntary effort of attention, but yet continue to play, just as a glare of light intently gazed upon will still be visible though we shut our eyes.

The poor king bewailed the vigil of his crowned head, and envied the mental fallow and slumber of the shipboy on the mast.

"The less men are raised above animal life," writes Southey, "the sounder the sleep is, and the more it seems to be an act of volition; with them, when they close their eyes, there is nothing to keep them waking."

It is vain then for us to try to sleep in this state, for the chain of thought cannot be broken.

> "My slumbers, if I slumber, are not sleep,
> But a continuance of enduring thought,
> Which then I can resist not."

Such was the penalty of Paganini's genius. He said, himself, that he seldom knew what sleep was, for his passion for music was an all-absorbing spell.

Boerhaave also has recorded his own case of insomnium. He had been intently thinking from morn till night on a very deep subject: the consequent insomnium lasted six weeks, during which period he scarcely closed his eyes. This was attended by a state of nervous apathy, until pain indicated a return of sensibility.

Viota was a parallel, (as we learn from Zimmerman,) who, in his paroxysms of mathematical abstraction, often kept awake and ate nothing for three days and nights.

Scipio, during the siege of Carthage, did not sleep for six days and nights. We are informed by Mr. Lay, that the aborigines of China, the Meaou-tsze, are totally unmindful of sleep.

This state then is one of temporary derangement; if often or long indulged in, permanent delirium or insanity may ensue. The brain is intently occupied by its own morbid ideas, and external impression is either a cypher or a chaos. Such the delirium of Manfred.

> "In my heart there is a vigil, and these eyes
> But close to look within."

The peril to mind or life depends on the degree or duration of the cause. The Dauphin was thus murdered by the constant induction of insomnium by his tormentor. Even Damien said the greatest torment he ever endured, was the want of sleep thus inflicted.

We must not, however, measure this abstractedly: the comparative impunity with which intense thought and insomnium are borne by some minds is very surprising, as in the instance of Alfred, Napoleon, Frederick, John Hunter, Pichegru, Wellington.

These great men were also very easily roused, which Wilson Philip says is characteristic of the most healthy slumber.

We have hinted that the passive mind will bear insomnium with impunity. Good cites the case of a maniac who slumbered very lightly merely a quarter of an hour in the day, and yet reached his 73rd year in perfect bodily health. We must be cautious, therefore, not *always* to deem insomnium a symptom of fresh disease, or even likely to induce it.

The effects of insomnium are constantly written on the body. Anthony was one of the sleek-headed men, and "such as sleep o' nights." Cassius, whose restless thoughts kept him awake, had a lean and hungry look. The finale of this indisposition sooner or later may be anticipated. The hot palm, the parched lip, the glaring eye, the pallid cheek, the languid muscle, will alike characterize the midnight watcher and the midnight debauchee,—the latter being, of course, easily recognised by tremor of the hands, the bloated visage, and the moral degradation.

There may, however, be *partial* insomnium; not that alluded to in the Article, which is merely a *lighter form.* Certain faculties of the mind (our late friend Wigan would say one of the brains) may fall asleep while others wake, as they often seem to do progressively and gradually: of this we have proofs in the illusions, incongruity, indeed monomania of a dream. On sudden waking, too, there is often an incongruity of thoughts, as if there was a *series* of wakings, until, the whole intellect being restored, the jumble is arranged. Thus there may be a fallow of some faculties while others work, and so the mind is preserved : if *all,* however, are kept on the stretch, sooner or later varied degrees of derangement will ensue—

> " Some perishing of study,
> And some insanity."

The imaginative brain is the fatal gift, the *" don du ciel,"* of that *irritabile genus,* so closely allied to madness. That which is the fine frenzy, or the dream, in a sound brain, will be, in one soft, sensitive, or diseased, the delirium of insanity. Dr. Copland refers to cases of this insomnium ending fatally: of a dignitary of the church who died apoplectic: of a physician who became insane : of a gentleman who was attacked by phrenitis after protracted pervigilium ; and we might cite many others.

Insomnium is, therefore, a kind of *υστερον προτερον,* the cause and consequence of insanity. In one case, it saps the intellect and makes it mad: in the other, the mad mind not being exhausted by active thought, has no immediate need of sleep. This constitutes the difference between active and passive insomnium, the wakefulness of the thought*ful,* and the wakefulness of the thought*less.*

This erethysm of the mind is often seen in nervous child-bed women, lapsing into insanity. It is either medullary or membranous irritation, or subacute inflammation, yet often subsiding on antiphlogistic treatment; but too frequently, in a delicate and self-tormenting tissue, the phantom, like that of Frankenstein, rises up and destroys its maker.

The insomnium of incipient insanity is not esentially a melancholy state, not marked by a want of or longing for sleep: it is often revelled in, and indulged by the cheromaniac. This is the more perilous form, inasmuch as, like that of the vices of excess, its early path is strewn with flowers. The system seems satisfied with the very lightest repose: like Antæus, it is but to touch the couch, and the slumberer at once rises refreshed, starting up in a moment, when we think him in a fair way for sound slumber.

We can, however, trace its stealthy march, from simple erethysm to confirmed mania.

At the onset, it is marked by eccentric and peculiar *habits*, constantly repeated ; such as a picking of the fingers, biting of the nails, a favourite route or style of progression. A young officer, who displayed cheromania in excess, was constantly walking round and round a table in the drawing-room, and picking his fingers almost to the sound of his steps. At other times the patient will dwell for whole pages on the same subject, a little varied, perhaps ; as if a phantom were always before him ; just as the remorseful or perturbed mind will brood over and ring the changes on one idea. This state is evidently not always painful. An insidious or placid smile plays over the countenance : if requested to sit or repose, there is no wish or acquiescence to do so,—the action seems a safety valve to the irritability, deriving indeed from the brain, or leaving it at rest, or as if there was an instinctive aim to procure sleep by weariness. We know that the brain must be at rest in our sleep : thus, we often slumber in the morning after a restless night and an accumulation of sorrows : the brain becomes quiescent, especially if some monotonous morning sound diverts for a minute the previously brooding thoughts.

This state is marked by *sudden* impulses. A patient will rise abruptly after an hour's repose, and resolve on the most absurd and untimely actions and pursuits—a condition too often disregarded as a mere eccentricity. He should, however, be closely watched, especially if insanity be hereditary in the family.

The dawn, or first impulse of passionate love, is a state of cheromania : the *couleur de rose*, which it flings over existence is to a certain degree constantly illusive. The lover indulges in his vigil as his chief happiness : the scene, however, if long protracted, changes. Old Burton is full of quaint allusions to the insomnium of love. Chariclea, when she was enamoured of Theogines, "lay much awake and was lean upon a sudden." Euryalus writes to Lucretia, "Tu mihi et somni et cibi usum abstulisti." Dido was not exempt : "At non infelix anima Phenissa, nec unquam solvitur insomnos," &c., &c.

Unconsummated love then, becomes a disease, and its endurance may well be called a passion, and the cavalier servente of Italy, Patito.

As the poets of all ages have alluded to insomnium as a prominent sign of love sickness, so has the painter displayed the effects of sleeplessness and anorexia in his enamoured youth.

In this incipient stage of insanity, the most strange perversions of moral sentiment, feelings, and expressions are observed,—one of the most prominent being a marked and intense aversion to previously beloved objects. An *in*version of thought seems to come on, somewhat as we see in the *extremes* of religious mania, the unitarian becoming a rigid catholic, and so forth. Some sense or consciousness of former error or folly occurs, and then they desire *as far as possible* to get clear of the stigma. Monomania cannot reason *moderately*: mole-hills are mountains, and soon follows on real hyperæsthesia of the mind.

The pathology of sleep is a deep study: that of insomnium, the privative of slumber, with its consequences and prototypes, must be equally hypothetical. When, especially, we are alluding to the moral and metaphysical causes, we must proceed entirely without leading strings; we have no *demonstration* to prove or illustrate our conclusions. The hearts of others are prone to conceal the truth, and, if we reason or deduce from our own case or state, it is clear that we do so with a perverted judgment. The deep sources, the exciting causes of sleeplessness, may often be sought in the dark recesses of a sorrowing or vicious brain, as well as in the intellectual, though, perchance, not less perilous labour of the moral virtuous mind. In either case the *texture* of the brain, its power of resisting or enduring mental labour, will constitute an important point; for we believe the cerebral is more concerned than the spinal system in the physiology of sleep. The excito-motory system must be awake, for we draw up our leg if the foot be tickled, but the memory retains no cognizance of it. If it so chance that extreme temptation has subdued to evil courses a mind, whose normal constitution was virtuous and good, the pang of remorse may be excited by a peccadillo,—the sensitive spirit broods over its delinquency, and the climax may be fatal. If the child of genius possess not a brain of firm and energetic texture, intellectual labour will not be endured without a morbid change, the prominent symptom of which will be insomnium, often lapsing into a protracted phantasy or delirium, which, accumulating in its course, will end often in disorganization of the encephalon.

The proximate cause of sleep has been ever a *questio vexata*. Depressed nervous energy, exhausted irritability, congestion in the cerebral sinuses, afflux of blood into the pia mater, its reflex towards the heart, deposition of fresh matter in the brain, cerebral collapse, deficiency of animal spirits, *vapor quidem benignus*;—these, and many other hypotheses, may be merely convertible terms, and they *explain* nothing.

That the circulation, quoad *quantity*, is influenced during sleep, we have had even ocular proof: the woman of Montpelier, whose case is recorded by Caldwell, had lost part of her skull, the brain and its membranes lying bare. When she was in deep or sound sleep, the brain lay in the skull almost motionless; when she was dreaming it became elevated; and when her dreams (which she related on awaking) were vivid or interesting, the brain was protruded through the cranial aperture. Blumenbach also witnessed a sinking of the brain during sleep, and a swelling with blood when the patient awoke.

The approach of sleep is marked by those phenomena which tend to diminish the action of the heart, and consequently of the circulation to the brain, and of all functions associated with the circulation.

Then as to *quality*: the varied phenomena of mind are constantly dependent on the crasis of the blood. The unhealthy state of the liver and other organs will indirectly affect the general circulation; every part of the system, of course, partaking of its influence, and every function being more or less deranged. The "influence of black blood on the brain" was made an especial subject in the "Philosophy of Mystery," several years ago, by Mr. Dendy. Dr. Binns has since referred to the point in his work on sleep. The subject, however, was fully discussed in the former work, and unacknowledged in the latter. When the normal or vicarious depurations of the system are interrupted or in abeyance, the brain will soon suffer, and its vessels assume a diseased action. Urea, carbon, or other poisons, will speedily show their influence on the brain.

We may glance, too, at the effect of artificial contamination. This is the record of Dionis, on referring to the first introduction of transfusion of brute blood into human veins:—"*La fin funeste de ces malheureuses victimes de la nouveauté, detruisit en un jour les hautes idées qu'ils avoient conçues; ils devinrent foux, furieux, et moururent ensuite.*"

Without asserting, then, that there is any specific vascular action, the crasis of the blood is a most important pathological point in reference to our subject. It must be evident to all who reflect on the rapidity with which psychical changes advance or recede. We may adduce also indirect evidence of unhealthy blood, in the odour and unctuous state of the skin in the sleepless idiot and lunatic. The excretion may be a sort of safety valve to the system or the brain, and indeed we may almost calculate on the degree of derangement from its excess.

The immediate effect of mental emotion of which we are conscious is on the heart. One prominent sign of cardiac derangement is insomnium, from the intimate sympathy, the direct intercommunication, indeed, of the heart and brain. There is no newly excited thought without an immediate impulse of the heart, so slight or transient perhaps, as not to be noticed. A sound, novel or unusual, will tend to keep the mind awake; but if this sound be oft repeated, so as to become familiar to the ear, then it does not *excite* the heart and brain, but rather *tends to sleep:* the secret, probably, of that mental repose and slumber amidst the loudest and most discordant sounds. Some sleep, indeed, seems to be produced by noise and excitement, but the terms are not convertible : monotonous sound is *not* excitement, but a *sedative.* Thus we sleep on a coach during the monotony of its rumbling and its motion : if these suddenly cease, we awake. But let the stoppage be protracted and permanent, that is, monotonous, we still sleep. The bellringer of Notre Dame found his lullaby in the loud ticking of the clock.

Now, if we may not consider the brain as a gland, secreting a thought or notion, at least it is the organ through which that thought is manifested. The idea, then, of an *action* in the brain is as clear as that of an action or function in a secreting gland, and we reason on its extreme derangements, such as insomnium or somnolency, as on enuresis or on jaundice. And this action obeys the laws of organic life ; if thought be in excess, the brain is exhausted, and hence disorder, disease, disorganization.

The immediate rush of scarlet blood to the brain is consequent on cardiac excitement : the first effect of this *determination* will be starting, agitation, exaltation of sense, especially hearing. Insomnium is the natural result of this *arterial* plethora or hyperæmia of the *systemic* heart.

Wardrop observes that "it is one of the most distressing symptoms which often accompany a disordered heart." And, again, "Those afflicted with disturbance of the heart suffer various imperfections of sleep. When in a profound sleep they sometimes start up in bed, completely awake, and are obliged to remain in the erect position in order to relieve a sense of impending dissolution. They are also subject to frightful dreams." Soon follows, usually, congestion, venous congestion or plethora of the *pulmonic* heart, and then the train of somnolency or intellectual oblivion comes stalking on—stupor, coma, apoplexy, death.

Somnolency or lethargy, however, in a pathological sense, is more allied to waking than to sleep, of which somnambulism and the dream are illustrations.

Hypertrophy and mitral disease, however, seem to induce contrasted effects on brain sleep, and consequently on varied degrees of insanity. Eccentric hypertrophy is the forerunner of cerebral excitement, inflammatory affections of the brain and its membranes. Concentric hypertrophy, inasmuch as the ventricle cannot contain the returning blood, and also mitral disease, as all other states which tend to derange or arrest the upper circulation, by pressure on the brain or cord, are constantly the remote causes of insomnium, or disturbed sleep. In sleepless maniacs we frequently observe the helix inflamed and tumid, and the eyes blood shot. The relief of the brain from the escape of blood, and consequently of stupor, insomnium, and even recent or transient insanity, is often evident. Epistaxis, hæmorrhoidal flow, or even the gush from an artery on the attempt at suicide will often at once restore sanity to the mind.

Analogous to these moral causes of the heart's increased action, are the mesmeric *passes:* for flushes and heat constantly precede the trance. This trance is not sleep: if *that* occur, the occupation of the mesmerist would be at once gone; it is the result of that congestion, which, like the effect of a brooding sorrow, is monotonous and all absorbing, and of the distraction of the mind from all else which would, through eye or ear, pass into the brain.

As sleeplessness is produced by, so it may, in its turn, induce heart-disease, by the *reaction* consequent on protracted pervigilium: hence, indeed, we shall have, as Copland observes, "more or less special influence upon the brain, heart, lungs, liver, &c., according to the susceptibility or predisposition of these organs."

The hypnotist, who asserts his never failing power of producing *sound sleep at will,* is guilty of gross ignorance, or extreme presumption: for hypnotics or the remedies of sleeplessness must be varied according to their varied causes, all, however, being concentrated to one end, the repose of the mind.

If we completely understood the essence or rationale of sleep, we might be able, by reproducing that, to ward off or overcome its antithesis; but our psycho-physiology is not perfect enough to determine the seat of the faculties, so as to enable us to attack the malady, or that one or more of these faculties which, being disturbed or still prone to work, *will not sleep.*

We may, perhaps, hope, if phrenology is ever fashioned into a system, to decide on the *seat* of a faculty, and if it be disordered, morally or physically, to restore it by some local remedy on or near its seat.

We seem, indeed, to be somewhat progressing, when we can fairly locate mind in the hemispherical ganglion, and consciousness in the cerebral base. This may be, in a degree, illustrated by the effects of situation or relative posture of the head, by which the principle of gravitation acts on the circulation; increased impetus, arterial plethora or venous congestion, inducing, by stimulation or pressure, all the phenomena of sleep, insomnium, and their affinities. When, therefore, the term *neurypnology* is used by Mr. Braid, the circulation must still be deemed the most essential point in the phenomena.

The interposition of the dura-matral processes between the brains must be remembered in our adoption of the position of the head. Hyperæmia of one portion of the encephalon, and anæmia of another, may thus be induced. Now, if all the organs of the brain were at once stimulated by scarlet blood, perfect insomnium would be the result, a state, probably, of extreme cheromania: if only a certain number, then we shall observe *relative* phenomena, various illusions, for instance, or eccentric actions. For these special irritations we refer to the lectures of Dr. Symonds, of Bristol, which we reviewed in our preceding number. The remedies for sleeplessness may be stated to be either moral, those which act on intellect or passions: physical, those which act through the system; psychical, those which influence the senses.

In referring to the requisites for sleep, the annotator of Hippocrates has thus written close up to this mark: "*tribus opus est ut quis placide dormiat, cerebro temperato, vapore benigno, et animo quieto.*"

The young psycho-pathologist, according to his metaphysical or organic learning, will be prone to look only on one side of the shield. We must, not, however, implicitly rely solely on moral suasion, or on physical remedy, but adopt a combination of the two; as brain congestion may be symptomatic of heart disease, or the immediate result of mental influence.

It will be our duty not only to *change matter*, but to regard that *something*, ethereal or spiritual, of which we have, at least, internal evidence, and which, by its more healthy alliance with brain may induce salutary results.

Without entering deeply into the reaction of mind and matter, we may observe that thought—self multiplying thought, on a *right* theme, might almost instantaneously change the ganglial molecules: and thus courtesy and kindness, and other psychical anodynes, might eventually weed "the bosom of that perilous stuff" that had poisoned it, and then the seeds of health may be sown on the mental soil with profit.

In our practice we have been constantly convinced of the influence of well-guarded conversation. We do not mean the doling out of a formal homily, but the cautious and placid allusion, even to the wanderings of the patient, at one time yielding or coinciding, at another explaining, in a familiar and cheerful way, so much of the cause as the patient can easily comprehend, or can placidly endure. The sleepless brain may thus be often soothed to slumber.

And this especially at the onset of insomnium, so often the incubation of insanity; for, as we have hinted regarding more severe degrees of mental disturbances, the chance of cure is in proportion to the brevity of the existence of insomnium. The germ of insanity may be thus blighted as it begins to expand.

It is not essential that we should here offer long comments regarding remedial agents; but we must remember that there is a variety of remote causes of brain excitement, and of consequent insomnium, which we must take the premonitory step of removing, ere we may hope to correct a habit or relieve a symptom.

Such are the various organic lesions. For hepatic engorgement, dyspepsia, lodgment in the cells of the colon, ascarides, the remedies are obvious ; mercurials, taraxacum, antacids, bitter purgatives, friction, exercise, and the habit of lying during digestion on the right side, or a frequent shifting of the body. The removal of many of these causes, will, by relieving pressure on the heart and lungs, engender brighter thoughts, a shadow will pass from the spirit, and slumber will ensue. The suppression of cutaneous and renal secretions, as well as latent or undeveloped gout, may induce a certain metastasis (?) to the brain, which may be cited as a cause of insomnium. The irritation of acute or inveterate skin disease, especially prurigo, and lichen, and other forms marked by hyperæsthesia of the skin, may be also adduced. These torments, especially when they occur during the state of pregnancy, become constantly aggravated towards *sleeping time.* Such sufferers should be allowed to slumber at any time when the subsidence of pruritus will allow them. The varicose condition of the veins of pregnant women, is sometimes followed, even after parturition, by a most uneasy state, which renders their nights sleepless. The relaxed valves and venous coats may be relieved by bandage and spirit lotions.

In all cases where hyperæmia or congestion is apparent, the loss of blood is often a most valuable antecedent. After depletion, the use of mercurials becomes more certain and effective, that of anodynes is rendered more safe and potent. If bleeding be contraindicated, opiates, of which the most eligible are the acetate of morphia, or the black drop, may be combined with antimony or digitalis, a form which, by inducing diaphoresis and diuresis, as well as by lowering vascular action, will go far to obviate narcotism and other baneful effects of opium.

The endermic method of administering opium, is often of much value, powdered opium being strewed on an abraded surface, or smeared at the outlets of mucous canals. In referring to sedatives, it has been ingeniously suggested, that, in cases of insomnium, where the pupil is expanded, opiates are the most eligible; where it is contracted, belladonna. The asthenic insomnia are easily diagnosed ; of course blood must be saved, and the anodyne or soothing modes immediately adopted.

In this form, the combination of seemingly contrasted remedies is often most judicious. Intestinal torpor requires a stimulant cholagogue; a languid yet irritable circulation demands a tonic anodyne ; and it is true that we especially observe the advantages of steel and morphia, particularly on convalescence from the acute stage of a malady. The *narcotic* influence of opium, even in increasing and often repeated doses, which is indeed the most efficient mode, is entirely obviated by the combination of aperients, anodynes and tonics.

In the languid system—concentrated nutrition should be administered, the beverage consisting of sweetwort, or infusion of malt or hop.

Pure air should be breathed by those who sleep unsoundly. The position of the pillow should be regulated according to these two forms of insomnium, the sthenic and the asthenic. In the first, the head should be high; in the second, almost horizontal. In the first, the pillow should be covered with oil silk, especially if cold cloths or sponges are employed; in the second, it may be formed of thin flannel, and filled with hops, especially if the patient be in advanced life.

Regarding some of the mechanical inducements to sleep, we may be taught by nature or instinct. At the onset of slumber, we, often unconsciously, proceed to the adjustment of our position, in order to compose the body, and obviate the stretch and strain of muscle. This may, perhaps, be a second cause or result: the hemispherical ganglion and therefore thought, being quiescent, as well as sensation and consciousness, the spinal system is left to its instinctive and its reflex actions, (just as a paralytic limb is often excited to unconscious action more easily than a sound one); the motive apparatus is then obedient, and the limbs prepare for sleep.

But if the spinal system be *exhausted*, then we have insomnium, and a tendency to twitchings or *fidgets;* a symptom, indeed, which is the first induced by mesmerism, ere the dropping into the trance. The psychical remedies, those which act on the brain, not so much as an organ of thought or reasoning as a concentration and sympathy of the senses, have long been made the subjects of mere morbid curiosity by scientific enthusiasts, and of mercenary extortion by the empirical hypnotist. They are all based on the principle of monotony. A prosy speech or sermon, the ticking of a clock, the hum of bees, the cawing of rooks, the plashing of the waterfall, the repetition of the alphabet, the counting of a thousand, protracted silence, the lullaby of the nurse, darkness—all influence the brain through this principle, and they are thus summed up by Spenser:—

> " Whiles sad night over him her mantle black doth spred,
> And more, to lull him in his slumber soft,
> A trickling streame from high rocke tumbling downe,
> And ever-dringling rain upon the loft,
> Mix'd with a murmuring winde, much like the soune
> Of swarming bees, did cast him in a swoune."

The mode adopted by the late *Hypnologist*, Gardner, consisted in fixing the attention, by listening to and counting one's own breathing: and this auricular hypnogeny was proved to be efficacious in the cases of many a well-known genius. The hypnogenic process of Mr. Braid is ocular, counting chiefly on the intense concentration of vision to one point.

When this monotony is combined with agreeable sensation, the effect will be more decided. Dr. Elliotson refers to a lady who sank into slumber whenever her husband rubbed her feet; and the animal magnetism combined with the luxurious abandonment of true affection will, *a fortiori*, induce the same happy slumber.

When a part is richly endowed with nerves, or possesses, naturally, very high sensibility, a slight electric effect seems to be induced by friction: the combing of the hair, especially that on the occiput, will constantly exert an anodyne influence, and we have no doubt that, in the state of hysterical insomnium, gentle friction of the areola of the mamma would often induce a disposition to slumber. We might here refer to the biological phenomena which have excited so much wonder and credulity; we might tell how Dr. Simpson sent a person to sleep, and commanded her not to wake for thirty-five hours; but we have before commented on these processes, which are all based on the abstraction of the mind from the thoughts or persons, the consciousness of which alike interferes with repose. A thought, an eidolon, or a person, will equally induce an action in the cerebrum which may be the exciting cause of insomnium.

We have thus briefly and discursively commented on a subject of deep importance, as well to the comfort of mankind as to our preservation from various psychical maladies. Our propositions, drawn from experiment and observation, will tend to complete the subject of sleep and sleeplessness, the *physiology* of which we had discussed in former essays. We have waived the recital of cases and anecdotes, many of which might have been familiar to our readers. We profess not to merit the full benediction of Sancho, for the *invention* of sleep, but we may hope, that we have contributed somewhat to insure or induce for many a careworn and sleepless being, the most exquisite balm of slumber.

